# Data on root system architecture of water efficient maize as affected by different nitrogen fertilizer rates and plant density

**DOI:** 10.1016/j.dib.2020.105561

**Published:** 2020-04-18

**Authors:** Abidemi Ruth Adebayo, Funso Raphael Kutu, Erick Tshivetsi Sebetha

**Affiliations:** a Food Security and Safety Niche Area Research Group, Faculty of Natural and Agricultural Sciences, North-West University Mafikeng Campus, Private Bag x 2046, Mmabatho 2735, South Africa; b School of Agricultural Sciences, University of Mpumalanga, P/Bag X11283, Mbombela 1200, South Africa

**Keywords:** Root system architecture traits, Shovelomics score board, Brace root angle, Crown root number, Planting season

## Abstract

Root system architecture is a resource with untapped potential for agricultural improvements. The presented data describes the root system architecture of water efficient maize lines to different nitrogen fertilizer rates and plant density at two locations in North West Province of South Africa. The experiment was carried out during the 2015/16 and 2016/17 planting seasons. The root system architecture traits brace root angle, brace root number, brace root branch depth, crown root angle, crown root number, crown root branch depth and number of lateral roots were scored with the aid of shovelomics score board. ANOVA was used to analyze the data set and means separated with DMRT (*p* ≤ 0.05).The regression analysis was used to determine the relationship among nitrogen fertilizer and root architecture system.

Specifications table

**Table:** 

Subject	Agricultural and Biological Science: Agronomy and Crop Science
Specific subject area	Plant physiology, plant biology, plant breeding, crop nutrition and soil fertility
Type of data	Table
	Figure
How data were acquired	Root system architecture were assess using shovelomics score board
Data format	Raw data
Parameters for data collection	Root system architecture was assessed at tasseling and physiological maturity stages using two uprooted plants from the based at 30 cm in each plot. The root system architecture traits brace root angle, brace root number, brace root branch depth, crown root angle, crown root number, crown root branch depth and number of lateral roots were scored with the aid of shovelomics score board.
Description of data collection	Root system architectures was assessed using a shovelomics score board
Data source location	The experiment was carried out at the Molelwane, North-West University (NWU) Research Farm (25° 48^1^S, 45° 38^1^ E.; 1012 m asl) and Taung Experimental Station (27 30^1^S, 24 30^1^E; 1111 m asl) of the Provincial Department of Agriculture Research Station during the 2015/2016 and 2016/2017 planting seasons. Both sites are located in the North West Province of South Africa.
Data accessibility	Raw data are attached as supplementary file.

## Value of the data

• The data showed the effect of different nitrogen fertilizer rates and plant density on root system architecture
• The data revealed the effect of soil types of each location on root system architecture.
• The data indicated the effect of interaction of nitrogen fertilizer rates, plant densities and locations on root system architecture.
• The data can be used by plant physiologist, plant breeders, crop nutritionist and general agronomist.

## 1 Data description

**Fig. 1:**
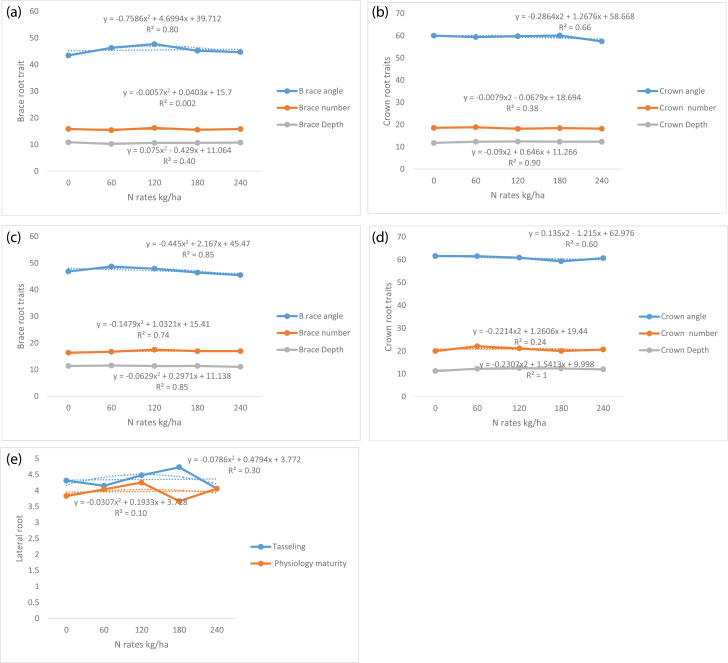
a. Regression relationship between N fertilizer and brace root traits during tasseling stage. b. Regression relationship between N fertilizer and crown root traits during tasseling stage. c. Regression relationship between N fertilizer and brace root traits during physiological maturity stage. d. Regression relationship between N fertilizer and crown root traits during physiological maturity stage. e. Regression relationship between N fertilizer and numbers of lateral roots during tasseling and physiological maturity stages.

The data describes the root system architecture of water efficient maize as affected by different nitrogen fertilizer rates and plant density in two locations of North West Province of South Africa. The experiment was carried out during 2015/16 and 2016/17 planting seasons. The meteorological data of experimental locations (Table 1). Tables 2–4 shows effect of each treatment factors (location, plant density and nitrogen fertilizer rates) on root system architectural trait. The interaction effect of location, plant densities and nitrogen fertilizer rates on root system architectural trait is presented in Tables 5 and 6. Table 7 presents relationship between architectural root system traits and grain yield. Fig. 1a–e presents relationship between N rates and root system architectural trait.

**Table 1:** The meteorological data of experimental locations.

	2015/16 planting season		2016/17 planting season		2015/16 planting season		2016/17 planting season	
	Molelwane Trial				Taung Trial			
Months	Temperature (°C)	Rainfall (mm)	Temperature (°C)	Rainfall (mm)	Temperature (°C)	Rainfall (mm)	Temperature (°C)	Rainfall (mm)
December	27.70	31.20	25.10	117.20	28.6	9.00	27.10	145.6
January	26.30	62.80	23.10	147.80	27.6	85.00	23.80	241.60
February	27.10	18.60	22.30	282.80	27.6	15.20	23.60	155.40
March	23.60	79.40	21.60	21.00	24.1	37.60	22.60	13.00
April	21.00	37.80	19.10	77.60	20.4	61.80	18.30	42.60
May	15.90	17.20	15.60	0.00	15.7	22.60	15.20	0.60
June	13.60	10.40	14.30	0.00	13.3	0.00	12.40	0.00
Total Mean	22.17	36.77	20.16	92.34	22.47	33.03	20.42	85.54

**Table 2:** Effect of treatment factors on brace root traits.

Treatments	Tasseling stage			Physiological maturity		
Location	B race angle	Brace number	Brace Depth	B race angle	Brace number	Brace Depth
Molelwane	45.27b	15.17b	7.79b	46.33b	16.55b	8,00b
Taung	45.67a	16.35a	13.92a	47.83a	17.41a	14.67a
LSD (*p* ≤ 0.05)	0.29	0.23	0.49	0.93	0.16	0.08
Plant density (plants/ha)						
33,333	46.16a	15.94a	10.19a	47.36a	16.43c	11.33b
44,444	44.06b	15.86a	10.94a	47.33a	17.39a	11.44a
55,555	46.17a	8.00b	10.64a	46.54a	17.13b	11.25b
LSD (*p* ≤ 0.05)	0.23	0.29	0.76	1.13	1.55	0.09
N rates (kg/ha)						
0	43.42e	15.83b	10.83a	46.87b	16.33c	11.35b
60	46.31b	15.42c	10.21b	48.69a	16.71bc	11.56a
120	47.68a	16.21a	10.62a	47.90a	17.48a	11.36b
180	45.21c	15.52bc	10.62a	46.44b	16.94b	11.38b
240	44.71d	15.81b	10.73a	45.48b	16.94b	11.04c
LSD (*p* ≤ 0.05)	0.37	0.37	0. 30	1.46	0.25	0.12

*Notes:* Means with the same letter(s) in the same column are not significantly different at *P* ≤ 0.05 according to Duncan's multiple range test.

**Table 3:** Effect of main treatment factors on crown root traits.

Treatment	Tasseling stage			Physiological maturity		
	Crown angle	Crown number	Crown Depth	Crown angle	Crown number	Crown Depth
Location						
Molelwane	61.72a	20.05b	12.21a	63.21a	20.96	12.08a
Taung	56.93b	23.57a	12.21a	58.44b	20.57	12.08a
LSD (*p* ≤ 0.05)	1.13	0.41	0.31	1.27	2.70	0.33
Plant density (kg/ha)						
33,333	59.51a	18.40b	12.06a	60.98a	21.46a	11.94b
44,444	59.42a	17.70c	12.20a	60.69a	20.10b	11.94b
55,555	59.02a	19.11a	12.38a	60.81a	20.74a	12.38a
LSD (*p* ≤ 0.05)	1.38	0.50	0.38	1.55	0.31	0.41
N rates (kg /ha)						
0	60.02a	18.52a	11.77b	61.60a	19.98b	11.25b
60	59.37a	18.83a	12.30a	61.56a	22.10a	12.29a
120	59.73a	18.10b	12.40a	60.90a	21.15b	12.50a
180	60.06a	18.42a	12.30a	59.33b	20.02b	12.40a
240	57.42b	18.15b	12.30a	60.69a	20.68b	11.98ab
LSD (*p* ≤ 0.05)	1.78	0.64	0.49	2.00	4.26	0.53

*Notes:* Notes: Means with the same letter(s) in the same column are not significantly different at *P* ≤ 0.05 to Duncan's multiple range test.

**Table 4:** Number of lateral root of WEMA maize as influenced by experimental location plant density and nitrogen fertilizer rates at different growth stages.

Treatment factors	Tasseling	Physiology maturity
Location		
Molelwane	4.75a	2.24b
Taung	3.94b	5.70a
LSD_(0.05)_	0.24	0.22
Plant density (plants/ha)		
33,333	4.33b	3.90b
44,444	4.60a	4.21a
55,555	4.13b	3.81b
LSD_(0.05)_	0.29	0.27
N rates (kg/ha)		
0	4.31bc	3.83bc
60	4.15bc	4.04ab
120	4.48ab	4.25a
180	4.73a	3.67c
240	4.06c	4.06ab
LSD_(0.05)_	0.38	0.34

*Notes:* Means with the same letter(s) in the same column are not significantly different at *P* ≤ 0.05 according to Duncan's multiple range.

**Table 5:** Interaction effect of treatment factors on brace root traits.

N rates	Plant density	Tasseling stage				Physiology maturity stage							
		Brace root angle (°)		Brace root number		Brace root branch depth (cm)		Brace root angle (°)		Brace root Number		Brace root branch depth (cm)	
		Mole	Taun	Mole	Taun	Mole	Taun	Mole	Taun	Mole	Taun	Mole	Taun
**0**	33,333	46.38	43.38	14.88	16.38	10.00	10.00	43.62	48.12	17.13	16.25	11.25	10.63
	44,444	40.62	46.75	14.38	15.38	10.00	11.25	46.12	48.25	15.88	18.63	11.25	11.25
	55,555	38.25	45.13	16.75	17.25	10.62	11.88	43.75	51.38	15.13	18.00	11.25	11.25
**60**	33,333	42.62	46.25	13.75	16.88	9.38	9.38	46.88	48.25	16.88	18.00	10.63	11.88
	44,444	52.00	49.25	13.38	15.13	10.63	10.62	50.25	49.88	16.75	16.88	11.25	11.25
	55,555	41.12	46.62	15.75	15.63	10.00	10.62	50.62	46.25	16.88	14.25	11.25	10.62
**120**	33,333	50.37	41.75	15.50	16.75	8.75	11.88	47.50	46.75	16.88	19.00	11.88	10.63
	44,444	47.12	47.75	15.50	15.13	10.00	11.25	44.88	50.87	17.63	17.88	11.25	11.25
	55,555	45.62	41.75	15.88	18.50	10.63	11.25	46.13	51.25	16.50	17.00	12.50	10.63
**180**	33,333	46.62	46.00	16.13	15.70	11.25	10.00	46.75	44.88	18.00	15.00	11.88	11.25
	44,444	42.13	40.75	14.00	15.38	10.63	10.62	45.38	46.50	16.00	19.50	11.88	11.25
	55,555	48.12	47.63	14.63	17.25	10.63	10.00	42.00	53.13	14.88	17.75	11.25	11.25
**240**	33,333	45.62	45.62	15.50	16.13	10.00	10.00	49.50	43.12	16.38	17.25	11.88	10.00
	44,444	49.38	46.0	17.25	17.25	11.88	10.00	46.75	44.37	15.88	16.38	11.25	10.63
	55,555	43.00	43.38	14.25	14.50	11.25	11.25	44.75	44.38	17.50	16. 38	11.25	11.25
**LSD_(0.05)_**		3.35		0.90		1.61		3.58		1.19		1.81	

*Mole = Molelwane and Taun = Taung.

**Table 6:** Treatment interaction effect on crown root traits.

N rates	Plant density	Tasseling stage						Physiology maturity					
		Crown root angle (°)		Crown root Number		Crown root branch depth (cm)		Crown root angle (°)		Crown root Number		Crown root branch depth (cm)	
		Molelwane	Taung	Molelwane	Taung	Molelwane	Taung	Molelwane	Taung	Molelwane	Taung	Molelwane	Taung
**0**	33,333	64.75	55.62	19.25	19.75	12.50	12.50	64.87	58.25	20.63	19.25	11.88	12.50
	44,444	65.87	51.62	16.37	16.37	11.88	11.88	62.87	56.87	19.63	18.63	10.63	11.25
	55,555	64.12	59.37	22.37	16.75	11.25	11.25	66.13	60.62	19.88	21.88	10.63	11.25
**60**	33,333	61.25	57.12	17.25	18.50	11.88	11.88	66.87	55.25	25.25	22.13	12.50	12.50
	44,444	66.38	60.38	20.50	17.87	11.88	11.88	67.00	60.62	19.50	19.12	12.50	11.88
	55,555	57.50	56.12	23.62	14.87	12.50	12.50	63.62	56.00	22.75	40.75	12.50	11.88
**120**	33,333	62.25	59.25	22.12	18.62	12.50	11.88	61.12	58.12	20.25	20.12	12.50	12.50
	44,444	62.00	57.37	20.50	15.50	12.50	12.50	60.37	63.25	14.75	26.88	12.50	12.50
	55,555	60.50	57.00	18.37	15.00	12.50	12.50	62.12	60.62	22.75	21.00	12.50	12.50
**180**	33,333	61.88	61.88	21.37	17.25	12.50	11.88	62.25	58.88	22.50	20.12	12.50	12.50
	44,444	59.50	55.00	18.25	15.25	11.88	12.50	62.87	59.12	17.63	26.88	12.50	11.88
	55,555	63.37	62.50	17.75	16.75	12.50	12.50	59.75	60.88	22.63	21.00	12.50	12.50
**240**	33,333	61.25	50.00	20.00	14.50	11.88	12.50	65.00	57.87	24.38	16.25	12.50	12.50
	44,444	63.62	55.00	20.25	16.62	12.50	12.50	64.75	56.50	23.38	22.63	11.88	11.88
	55,555	58.37	56.37	19.62	17.87	11.88	12.50	65.75	54.25	19.00	19.62	12.50	11.25
**LSD_(0.05)_**		1.63		1.57		1.19		3.21		5.29		0.65

**Table 7:** Effect of location, plant density and nitrogen fertilizer rates on number of lateral root of WEMA at different growth stages.

N rates	Plant density	Tasseling stage		Physiology maturity stage	
		Molelwane	Taung	Molelwane	Taung
**0**	33,333	4.88	4.00	4.12	3.63
	44,444	3.25	4.88	3.75	3.13
	55,555	5.13	3.25	3.63	4.75
**60**	33,333	5.50	3.25	4.13	3.88
	44,444	4.13	3.50	4.13	4.13
	55,555	4.75	3.75	4.00	4.00
**120**	33,333	3.75	4.63	4.00	4.75
	44,444	4.13	4.38	4.00	3.75
	55,555	6.00	4.00	5.00	4.00
**180**	33,333	4.88	4.63	3.38	3.00
	44,444	6.25	3.50	4.25	3.13
	55,555	4.75	4.38	3.63	4.63
**240**	33,333	4.75	3.00	3.88	4.13
	44,444	4.13	4.75	4.13	3.75
	55,555	5.00	4.13	4.00	4.50
**LSD _(0.05)_**		0.47		0.43

**Table 8:** Relationship between root system architectural traits and grain yield.

Root architecture parameters	Physiology maturity stage	
	Equation	R^2^
Brace root angle	*y* = 0.0017×^2^ - 0.0577x + 2.8048	0.65**
Brace root number	*y* = 0.0287×^2^ - 1.0313x + 13.628	0.007ns
Brace root branch depth	*y* = 0.1003×^2^ - 1.6616x + 8.9689	0.65**
Crown root angle	*y* = 0.0048×^2^ - 0.4739x + 13.69	0.74**
Crown root number	*y* = −0.0018×^2^ + 0.36x - 2.0552	0.62**
Crown root branch depth	*y* = 0.0854×^2^ - 1.3006x + 6.9787	0.71**
Number of lateral root	*y* = −0.0515×^2^ + 1.3576x + 0.1385	0.56*

## 2 Experimental design, materials, and methods

### 2.1 Description of study area

The experiment was carried out at the Molelwane, North-West University (NWU) Research Farm (25° 48^1^S, 45° 38^1^ E.; 1012 m asl) and Taung Experimental Station (27 30^1^S, 24 30^1^E; 1111 m asl) of the Provincial Department of Agriculture Research Station during 2015/2016 and 2016/2017 planting seasons respectively. Both sites are located in the North West Province of South Africa. The experimental soils were Ferric Luvisol and Rhodic Ferralsol. The chemical properties of Ferric Luvisol are pH (4.41) total N (0.13%), available P (43 mg/kg) and K (241 mg/kg). However, the Rhodic Ferralsol had the following chemical properties, pH (5.38), total N (0.10%), available P (27 mg/kg) and K (207.5 mg/kg) across two planting seasons.

There were five N rates (0, 60, 120, 180 and 240 kg N/ ha) and three plant densities (33,333, 44,444 and 55,555 plants/ ha). The experiment was laid out in split plot and the treatments were arranged in randomized complete block design, replicated four times. The main plot effect was the three plant densities (33,333, 44,444 and 55,555 plants/ha) while the five N fertilizer rates (0.60, 120,180 and 240 kg N/ha) constituted the sub plot effect. Maize (WE 3127) seeds were sown at spacing of 1 m x 0.3 m, 0.75 m x 0.3 m and 0.9 m x 0.2 m to achieve the density of 33,333, 44, 4444 and 55,555, respectively. The fertilizer application treatment was carried out by applying a third of the each rate as basal treatment at planting using NPK 20:7:3 while two-third and a third of the remaining quantity from each rate was applied as top dressing at 3 and 5 weeks after sowing (WAS) using lime ammonium nitrate (LAN, 28%).Weeding was done manually at 3 and 7 weeks after sowing.

### 2.2 Assessment of root system architecture

Root system architecture was assessed at the tasseling and physiological maturity stages using two uprooted plants from the based at 30 cm in each plot. A manually designed shovelomics score board was used to score the root architecture as described by Trachsel et al. [2]. Root system architectural traits assessed include brace root, crown root and lateral root with focus on the number, branching angle and depth. Root depths were classified as shallow or deep/steep. Root with 0–5 cm depth was classified as shallow while that within 5–10 cm depth was classified as deep/steep as described by Trachsel et al. [2]. Classification of brace and crown angle was 10–50° as shallow and 50–90° as deep and steep while assessment of root number was by counting using the standard procedure described by Trachsel et al. [2]. Grain yield of WEMA maize was obtained as described by Adebayo [1].

### 2.3 Statistical analysis

All data obtained were subjected to analysis of variance (ANOVA) using the GenStat 11th edition. Differences between the treatment means were separated using Duncan Multiple Range Test (DMRT) test at 5% level of probability. Regression was used to estimate relationship between N rates grain yield and root system architectural trait using Excel program.

## Appendix Supplementary materials

Image, application 1

Image, application 2
